# Supplementary material for: Detailed molecular and epigenetic characterization of the pig IPEC-J2 and chicken SL-29 cell lines
Source: iScience. 2023 Feb 20;26(3):106252. doi: 10.1016/j.isci.2023.106252 (PMC10018572; doi:10.1016/j.isci.2023.106252)
Supplement: Data S1. Complete homer output for identified motifs in Pig IPECJ-2, related to Table 2 — Homer motif analysis results for histone modifications H3K4me1, H3K4me3, H3K27ac, and enhancer elements of pig IPECJ2 cell line. P-values >1e-10 are possible false positives. Within each folder (e.g. peak_files_CTCF) are the html files showing the identified motifs when using homer (e.g. homerResults.html). [file mmc2.zip › S5/Pig_IPECJ_2/peak_files_enhancer/homerResults/motif2.similar.html]

motif2

## Information for motif2

A
C
G
T
G
T
A
C
C
T
G
A
T
G
A
C
G
T
C
A
C
A
G
T
C
G
A
T
G
A
T
C
G
A
T
C
C
G
A
T
  
Reverse Opposite:  

G
C
T
A
C
T
A
G
C
T
A
G
G
C
T
A
G
T
C
A
C
A
G
T
A
C
T
G
G
A
C
T
C
A
T
G
T
G
C
A
  

|  |  |
| --- | --- |
| p-value: | 1e-177 |
| log p-value: | -4.098e+02 |
| Information Content per bp: | 1.583 |
| Number of Target Sequences with motif | 7086.0 |
| Percentage of Target Sequences with motif | 19.34% |
| Number of Background Sequences with motif | 5097.5 |
| Percentage of Background Sequences with motif | 13.94% |
| Average Position of motif in Targets | 148.5 +/- 83.4bp |
| Average Position of motif in Background | 150.0 +/- 86.7bp |
| Strand Bias (log2 ratio + to - strand density) | -0.0 |
| Multiplicity (# of sites on avg that occur together) | 1.13 |
| Motif File: | file (matrix) reverse opposite |

### Similar de novo motifs found

|  |  |  |  |  |  |  |  |
| --- | --- | --- | --- | --- | --- | --- | --- |
| Rank | Match Score | Redundant Motif | P-value | log P-value | % of Targets | % of Background | Motif file |
| 1 | 0.966 | C T G A G T A C C G T A C A G T C G A T G T A C G A T C C G A T | 1e-169 | -390.396067 | 25.03% | 19.12% | motif file (matrix) |
| 2 | 0.709 | T C A G T G A C G T C A A C G T A C G T A G T C G A T C G C A T C A T G A T C G A T G C G C A T A T C G | 1e-45 | -104.639454 | 7.51% | 5.71% | motif file (matrix) |
| 3 | 0.720 | C A G T A C G T A C G T A G T C C G T A T G A C C G T A A C G T G A C T G T A C G T A C G A C T A C G T | 1e-32 | -75.606857 | 0.31% | 0.08% | motif file (matrix) |
| 4 | 0.726 | C G T A A G T C C G T A A C G T C G T A A T G C A G T C C G T A | 1e-27 | -64.073597 | 4.00% | 2.97% | motif file (matrix) |
| 5 | 0.636 | C G T A A C G T A C G T A G T C A G T C C G T A A G T C C G T A A C T G C G T A | 1e-21 | -49.542357 | 0.21% | 0.06% | motif file (matrix) |
| 6 | 0.613 | A G T C C T G A C G T A A C T G A C T G C G T A A G T C A C G T C T A G A G T C | 1e-14 | -34.035008 | 0.17% | 0.05% | motif file (matrix) |
